# Supplementary material for: Cannabidiol Antiproliferative Effect in Triple-Negative Breast Cancer MDA-MB-231 Cells Is Modulated by Its Physical State and by IGF-1
Source: Int J Mol Sci. 2022 Jun 27;23(13):7145. doi: 10.3390/ijms23137145 (PMC9266539; doi:10.3390/ijms23137145)
Supplement: Supplementary file 1 [file ijms-23-07145-s001.zip › Supplementary Figures.pptx]

## Slide 1
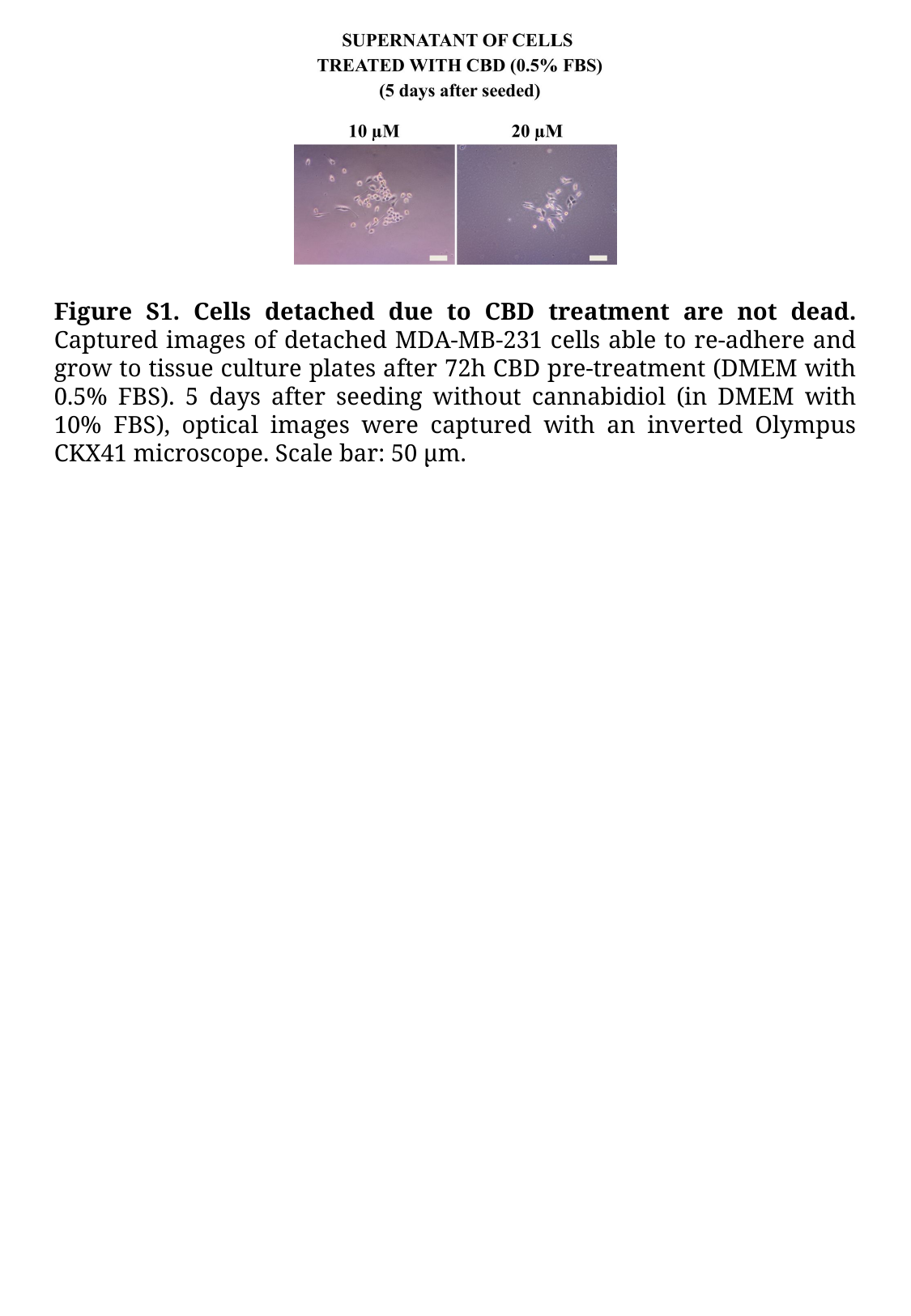

Figure S1. Cells detached due to CBD treatment are not dead. Captured images of detached MDA-MB-231 cells able to re-adhere and grow to tissue culture plates after 72h CBD pre-treatment (DMEM with 0.5% FBS). 5 days after seeding without cannabidiol (in DMEM with 10% FBS), optical images were captured with an inverted Olympus CKX41 microscope. Scale bar: 50 µm.

## Slide 2
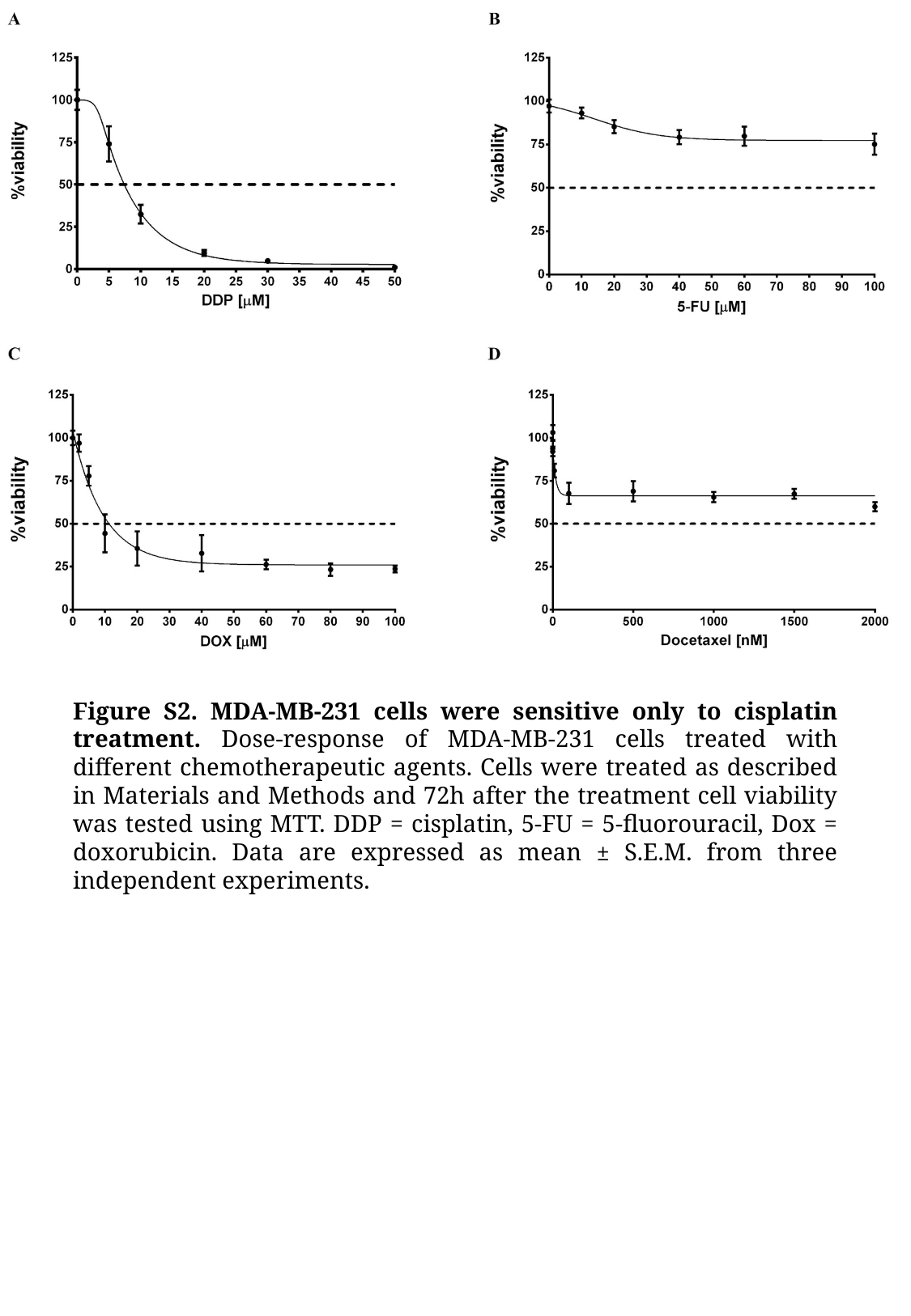

Figure S2. MDA-MB-231 cells were sensitive only to cisplatin treatment. Dose-response of MDA-MB-231 cells treated with different chemotherapeutic agents. Cells were treated as described in Materials and Methods and 72h after the treatment cell viability was tested using MTT. DDP = cisplatin, 5-FU = 5-fluorouracil, Dox = doxorubicin. Data are expressed as mean ± S.E.M. from three independent experiments.

## Slide 3
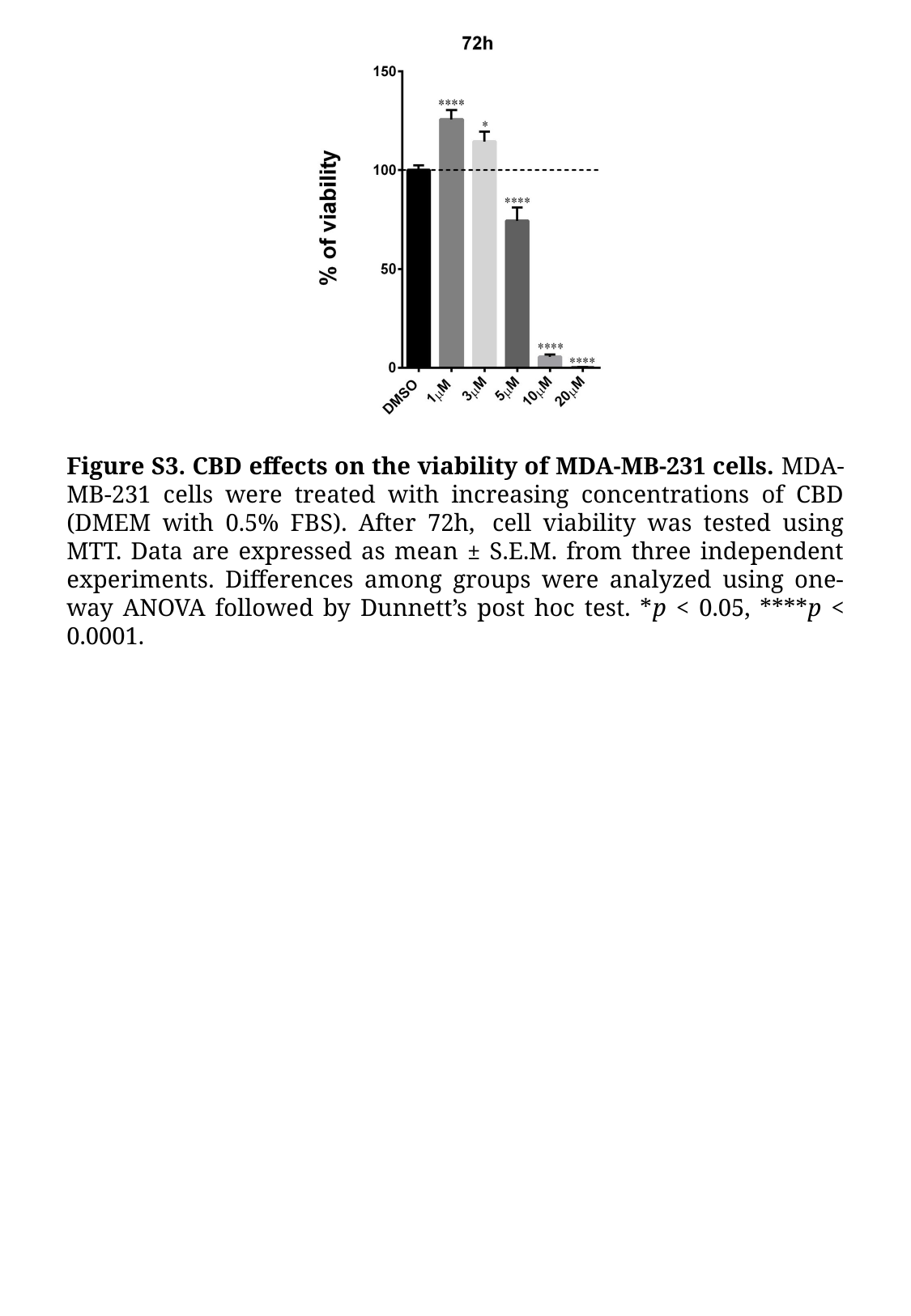

Figure S3. CBD effects on the viability of MDA-MB-231 cells. MDA-MB-231 cells were treated with increasing concentrations of CBD (DMEM with 0.5% FBS). After 72h,  cell viability was tested using MTT. Data are expressed as mean ± S.E.M. from three independent experiments. Differences among groups were analyzed using one-way ANOVA followed by Dunnett’s post hoc test. *p < 0.05, ****p < 0.0001.

## Slide 4
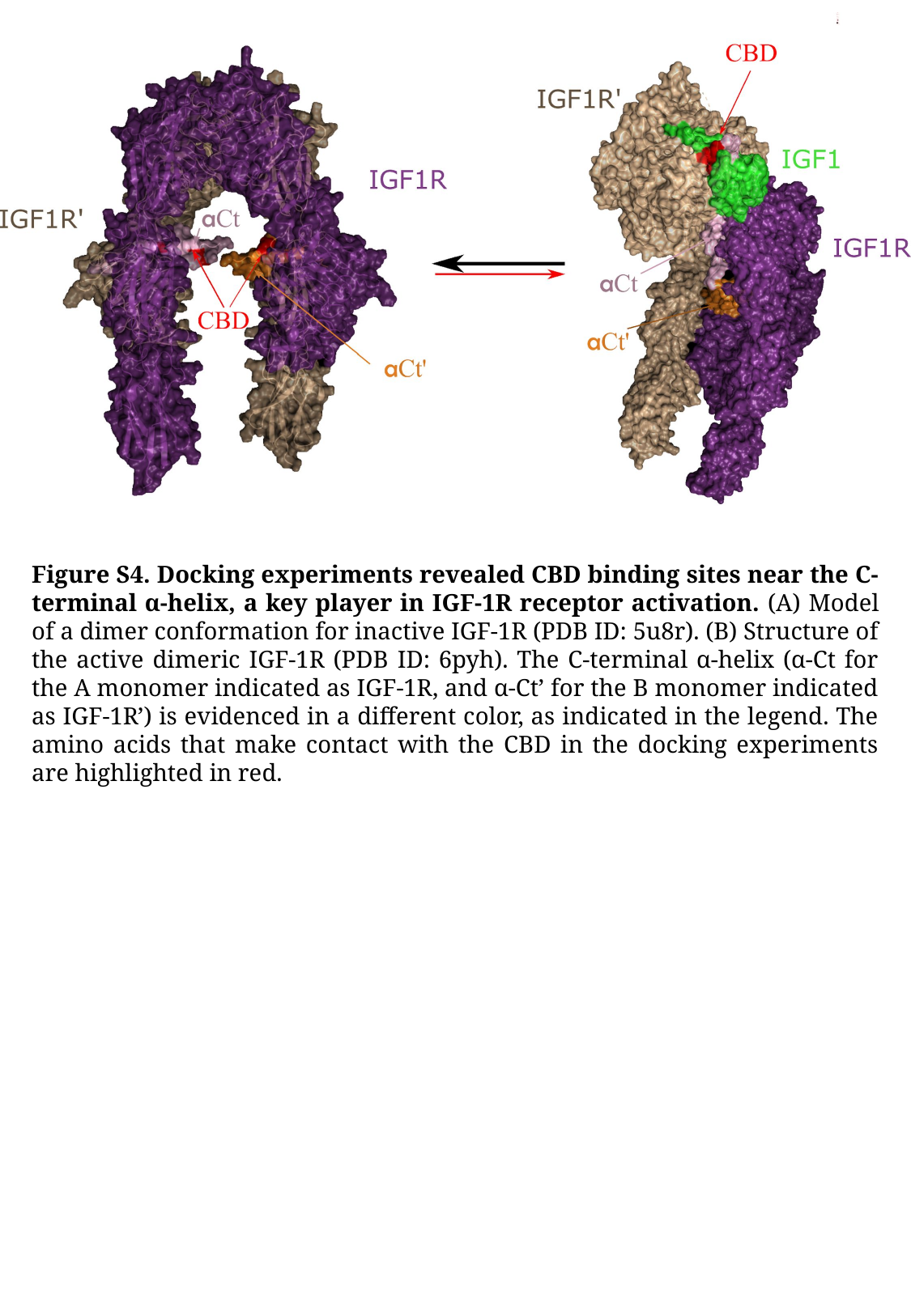

Figure S4. Docking experiments revealed CBD binding sites near the C-terminal α-helix, a key player in IGF-1R receptor activation. (A) Model of a dimer conformation for inactive IGF-1R (PDB ID: 5u8r). (B) Structure of the active dimeric IGF-1R (PDB ID: 6pyh). The C-terminal α-helix (α-Ct for the A monomer indicated as IGF-1R, and α-Ct’ for the B monomer indicated as IGF-1R’) is evidenced in a different color, as indicated in the legend. The amino acids that make contact with the CBD in the docking experiments are highlighted in red.

## Slide 5
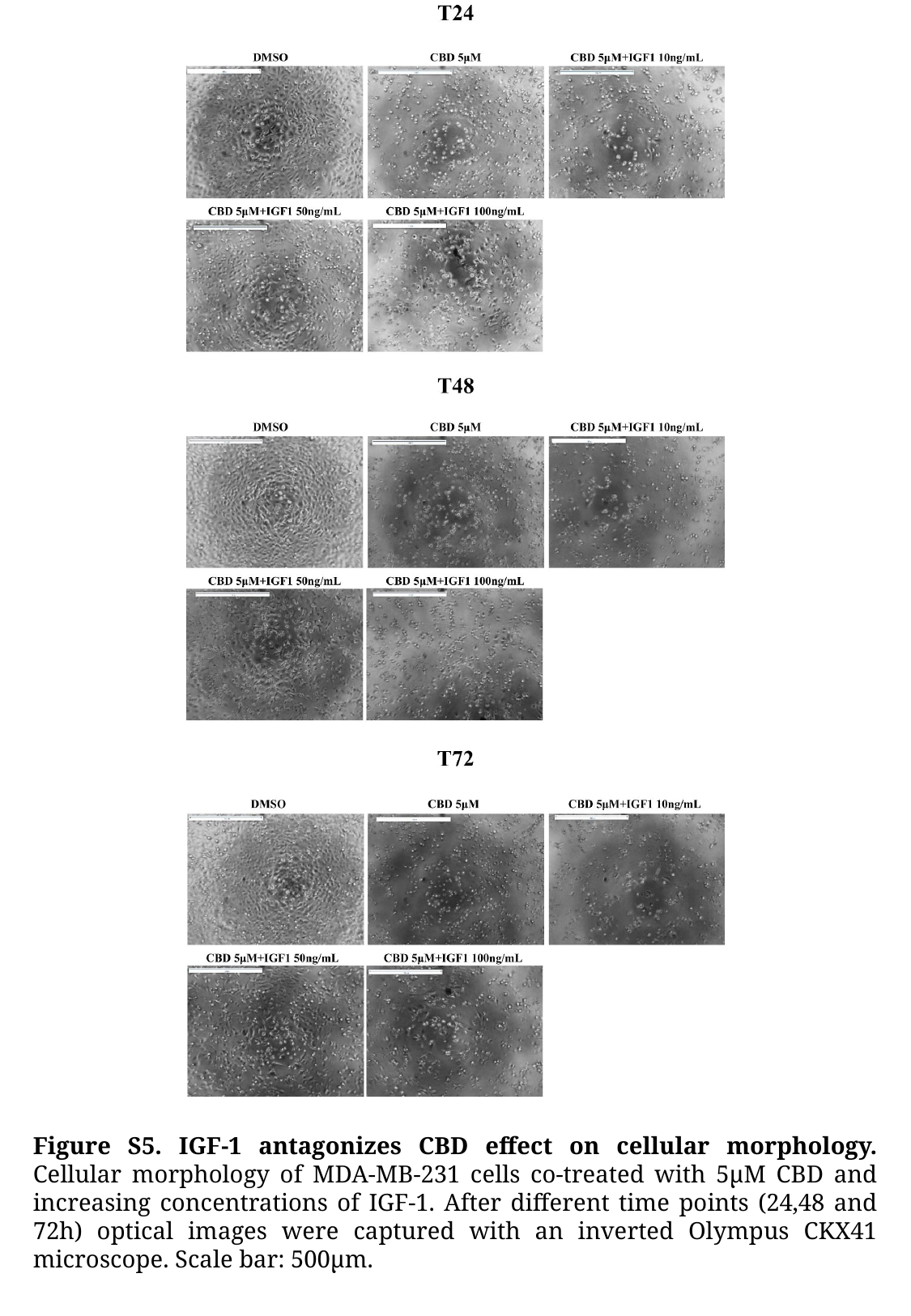

Figure S5. IGF-1 antagonizes CBD effect on cellular morphology. Cellular morphology of MDA-MB-231 cells co-treated with 5µM CBD and increasing concentrations of IGF-1. After different time points (24,48 and 72h) optical images were captured with an inverted Olympus CKX41 microscope. Scale bar: 500µm.
